# Life on the Line: Prioritizing Equity in Kidney Transplantation for Populations Marginalized by Race and Ethnicity

**DOI:** 10.1177/20543581251331076

**Published:** 2025-04-10

**Authors:** Jagbir Gill, Reetinder Kaur

**Affiliations:** 1 Division of Nephrology, The University of British Columbia, Vancouver, BC, Canada; 2 Interdisciplinary Studies Graduate Program, The University of British Columbia, Vancouver, BC, Canada

**Keywords:** Kidney transplantation, access to transplantation, disparities

Nearly 45% of all Canadians with kidney failure are living with a functioning kidney transplant.^1^ However, the life-altering benefits of kidney transplantation are not accessible to all Canadians. Populations marginalized by race and ethnicity have reduced access to transplantation across Canada.^2-11^ First Nations, Inuit, and Métis patients are 66% less likely to receive kidney transplantation compared to white Canadians and experience longer wait times, despite having a threefold greater risk of kidney failure.^3^ Similarly, African, Caribbean, and Black (ACB) Canadians are 65% to 69% less likely to receive living donor kidney transplantation^4,12,13^ and East Asian Canadians with kidney failure are 29% and 73% less likely to receive kidney transplantation and living donor kidney transplantation, respectively.^8,11,14^ South Asian Canadians have a 50% to 77% lower likelihood of receiving a transplant^4,8-11^ and are also less likely to identify even a single potential living kidney donor.^15,16^ These disparities are not confined to Canada; similar inequities have been reported among these and other communities worldwide.^3,17^ However, despite decades of descriptions outlining these patterns, the reasons underlying these disparities remain incompletely understood.^5,11^

Sociodemographic inequities in access to transplantation are likely a result of a complex interplay of intersecting realities and identities, including race, age,^2,7,18,19^ gender,^19,20^ geographic isolation,^4,6,19,21,22^ socioeconomic status,^21,23^ cultural beliefs,^4,18,24^ religious beliefs,^4,18^ decision-making complexities,^5,25^ and systemic barriers including bias and racism.^18^ Key findings over the past decade have highlighted the challenges patients face when navigating the long, complex, and costly transplant evaluation process, with studies showing that racialized patients experience delays in referral, evaluation, and wait-listing for kidney transplantation.^26^ Socioeconomic status and geographic isolation are important intersecting factors that further impact access to transplantation, with lower income patients, disproportionately from racialized communities, and rural and remote patients, disproportionately from Indigenous populations, facing additional hurdles when accessing transplant.^27-29^ Similarly, gender-based disparities further amplify racial inequities, with racialized women disproportionately impacted.^4,11,30^ In addition, a lack of trust in the health care system, rooted in historical and ongoing evidence of culturally unsafe care,^31^ has been shown to deter patients from Indigenous and other patients marginalized by race and ethnicity from pursuing donation and transplantation.^29^ Therefore, addressing inequities warrants multifaceted interventions that incorporate sociologic, health system, and health policy components. These are difficult to study, implement, and evaluate.

Another important consideration when understanding and evaluating racial inequities is that each community is not homogeneous. Although the term “Indigenous peoples” in Canada collectively refers to three groups—First Nations, Inuit, and Métis—there exists considerable diversity within each group, as evidenced by linguistic (more than 70 Indigenous languages), cultural (more than 600 First Nations communities), and geographical variations.^32^ Similarly, despite the linguistic, cultural, and religious diversity within settler communities, many subgroups are often subsumed under overarching labels such as “South Asian” or “East Asian.”^8,11,24,25,33^ This is problematic, as existing research largely fails to adequately represent the distinct experiences of these subgroups,^33^ and this, in turn, may limit the specificity of culturally tailored interventions.

## Although Some Initiatives Highlight Potential Paths Forward, Much More Work is Needed

In a recent scoping review of health equity data in organ transplantation,^34^ randomized studies testing educational interventions and support services were highlighted as a *rare area* in equity research that has demonstrated efficacy in increasing access to kidney transplantation. Notably, health services interventions that have yielded mixed results in the general kidney transplant population have shown significant promise when tailored to specific patient populations marginalized by race and ethnicity.

A recent multicomponent cluster randomized clinical trial, EnAKT LKD, evaluated the impact of education and peer support for patients with chronic kidney disease in Ontario, Canada and did not demonstrate an increase in the rate of completed steps toward receiving a kidney transplant.^35^ However, this study was not targeted to address racial and ethnic disparities in access and did not include culture-specific interventions. In contrast, culturally targeted studies among African Americans^36-39^ and Hispanic Americans^40^ have demonstrated that culturally and linguistically appropriate educational interventions may lead to significant improvements in knowledge, patient attitudes toward transplantation, satisfaction with culturally congruent care, and pursuit of kidney transplantation, with some studies reporting significant increases in living donor kidney transplantation rates.^39^

Similarly, recent studies in the general kidney transplant population have suggested that patient navigators may have limited effectiveness in increasing the likelihood of transplantation,^37,41,42^ but race and culture-specific barriers have not been explicitly addressed in these studies, and there are little published data on the role of transplant navigators to address racial and ethnic inequities. However, experiences from non-transplant populations highlight the potential benefit of such an approach. Indigenous patient navigators have been effectively incorporated in cancer care models with evidence of improved participation in cancer screening^43^ and high levels of patient satisfaction.^44^ Notably, the role of navigators in supporting patients as an advocate through the health care system has often been a key component and the need to collaborate with communities and hospital systems to ensure smooth implementation has been highlighted.^44^

While Indigenous living kidney donors, kidney failure patients, and Elders have voiced the need for navigation supports,^29^ no published studies have evaluated this approach. The BRIDGE to Transplant initiative is an implementation study currently being conducted in British Columbia (BC) which aims to improve access to kidney transplantation for Indigenous patients. The study is evaluating the impact of patient and community informed interventions including culturally safe education, mandated early referral, Indigenous wellness liaison support, and navigation supports.^29^ The results of this work may provide insights into the feasibility and effectiveness of a culturally targeted strategy for both education and navigation.

In addition to patient targeted interventions, community-based engagement and research are critical to address transplant inequities. ACB organ health (http://www.youtube.com/@acborganhealth) is a community-led YouTube channel launched in response to recommendations from the Health Canada–funded ACTION project,^11,25^ which partnered with ACB and South Asian community members to understand inequities in access to transplantation in Ontario and BC. Launched in partnership with the University Health Network Centre for Living Donation in 2021, the channel is dedicated to sharing experiences and accessible knowledge of transplantation from ACB communities and works in partnership with ACB patient partners and actively engage the ACB community.

The Saskatchewan First Nations and Métis Organ Donation and Transplantation Network was incepted by Dr Caroline Tait in 2019 as an Indigenous think tank aimed to bridge Indigenous, biomedical, health care, policy, legal and research interests in organ donation and transplantation.^23^ This ultimately led to the development of an international consortium which includes Indigenous people with lived experience and knowledge keepers along with Indigenous researchers and non-Indigenous medical and research experts.^23^ This initiative has critically raised the profile of organ failure and transplantation in Indigenous communities across Canada, most notably evidenced by the recent passing of a resolution by the Union of BC Indian Chiefs to support improved access to treatment for organ failure, organ donation and transplantation for First Nations.^45^ However, given the number of competing health-related crises facing Indigenous communities across Canada, it is imperative that this work be expanded and appropriately funded to ensure community engagement on issues related to kidney failure and transplantation persist.^23^

Internationally, the National Indigenous Kidney Transplantation Taskforce (NIKTT) serves as an example of a coordinated and government funded organization that leverages partnership between communities and key health stakeholders. Established and funded as an initiative of Transplantation Society of Australia and New Zealand (TSANZ) in 2019, NIKTT aims to improve access to and outcomes of kidney transplantation for Aboriginal and Torres Strait Islander peoples. The initiative includes diverse stakeholders, such as Indigenous kidney community members, nephrologists, nurses, policymakers, researchers, as well as primary care and allied health professionals. National Indigenous Kidney Transplantation Taskforce is responsible for driving the development and implementation of key initiatives that address knowledge and service delivery gaps identified in the TSANZ Performance Report,^46^ including enhanced data collection and reporting and pilot initiatives to improve patient equity and access.^47^ Such initiatives offer significant potential and should be considered in the Canadian context.

## Advancing Equity in the Current Climate: A Call to Action

As we navigate the realities of a fiscally constrained health care system and witness staggering global shifts in sociopolitical support for diversity, equity, and inclusion initiatives, it is paramount that we are steadfast in our collective commitment to address inequities in access to health care. In the case of kidney transplantation, despite our longstanding recognition of inequities impacting patients who are Indigenous or marginalized by race or ethnicity, we have only just begun to invest in the work required to meaningfully combat this issue. As we strive toward equitable access to transplantation, all stakeholders need to work collectively and strategically to address the complex underpinnings of this issue.

To do so, we must:

– *Further partner with patients and communities.* Working in partnership with patients and communities ensures that our efforts are meeting the needs of target populations, raises awareness about organ donation and transplantation, and significantly widens the collective voice to advocate for equitable access to transplantation. Critically, this partnership needs to span the spectrum of research, policymaking, and governance, mandating inclusion of key patient and community stakeholders in each of these forums.
– *Have a national consortium address inequities in access to transplantation*. While respecting region-specific challenges, nuances, and considerations, it is imperative that we lend a national voice and coordinate our efforts to address inequities in transplant through which we can drive awareness, innovation, and meaningful, measurable change. We must learn from models, such as the NIKTT, to empower Indigenous voices and support shared knowledge and learnings by working with Indigenous health leaders and academics. Such a consortium may also support community-led educational and outreach programs to address community needs, increase transplant literacy, and dispel misinformation.
– *Participate in promotion of organ donation*. The organ shortage continues to be the dominant barrier to widespread transplantation and efforts to expand organ donation are paramount. In a post-pandemic world where trust in institutions and health care has been eroded, new approaches are needed to appeal to communities, particularly those with limited awareness of organ donation. As clinicians, academics, and advocates for patients with kidney failure it is within our mandate to participate in raising awareness for deceased and living donation.
– *Invest in health services initiatives that make it easier to navigate the transplant process*. Structural and systemic barriers to care are rooted in systems that may be outdated, culturally insensitive, and may propagate bias and racism. It is important that these systems be critically evaluated and modified to better meet the needs of our patients. Importantly, while health services intervention studies in transplantation have focussed on patient education and navigation with mixed results, these initiatives have often not considered the sociocultural and identify dynamics of the patient population. Therefore, more targeted, culturally tailored health services interventions that also aim to address intersecting factors such as financial and geographic barriers need to be evaluated. It is also important to recognize that engaging in education, support, and culturally safe care is a long-term investment to build trust, comfort, and improve the patient experience. In the context of tackling inequities, this is a critical long-term goal that warrants ongoing investment.
– *Prioritize data needs*. The paucity of readily available race-based data limits our understanding of disparities and breeds inaction. Recent investments by the Canadian government in our national organ donation and transplant data system has provided an opportunity to more robustly capture, analyze, and report on key metrics. It is critical that metrics to capture and report on inequities in transplantation are prioritized, while ensuring that principles of data ownership, control, access, and possession are maintained with Indigenous communities and populations marginalized by race or ethnicity.

Ensuring equitable access to kidney transplantation aligns with Canada’s broader health care equity objectives and represents an ethical imperative. Transitioning from acknowledging disparities toward actively addressing and dismantling them demands dedicated efforts, sustained collaboration, adequate funding, and targeted systemic reforms. The opportunity for change is clear, and the imperative to act is urgent.
